# Investigating the clinical, pathological and molecular profile of oncocytic adrenocortical neoplasms: a case series and literature review

**DOI:** 10.1530/EO-21-0011

**Published:** 2021-08-16

**Authors:** Eleanor Fewings, Serena Khoo Sert Kim, Alexey Larionov, Alison Marker, Olivier Giger, Ashley Shaw, Graeme R Clark, Vasilis Kosmoliaptsis, Benjamin G Challis, Marc Tischkowitz, Ruth T Casey

**Affiliations:** 1 Academic Department of Medical Genetics, National Institute for Health Research Cambridge Biomedical Research Centre, University of Cambridge, Cambridge, UK; 2 Department of Endocrinology, Cambridge University Hospital NHS Foundation Trust, Cambridge, UK; 3 Department of Histopathology, Cambridge University Hospital NHS Foundation Trust, Cambridge, UK; 4 Department of Pathology, University of Cambridge, Cambridge, UK; 5 Department of Radiology, Cambridge University Hospital NHS Foundation Trust, Cambridge, UK; 6 Department of Surgery, University of Cambridge and NIHR Cambridge Biomedical Research Centre, Cambridge University Hospitals NHS Foundation Trust, Cambridge, UK; 7 East Anglian Medical Genetics Service, Cambridge University Hospital NHS Foundation Trust, Cambridge, UK

**Keywords:** oncocytic adrenocortical neoplasms, malignant, Lin-Weiss-Bisceglia system, oncocytic tumours

## Abstract

**Background:**

Malignant oncocytic adrenocortical neoplasms (OANs) are rare tumours with a distinctive biological behaviour compared to conventional adrenocortical carcinoma (ACC). The current prognostic systems overestimate the malignant potential of these tumours, and guidance for surveillance and treatment strategies are lacking.

**Aim:**

To evaluate the utility of clinical, pathological and molecular markers in predicting the biological behaviour and outcomes of malignant OANs.

**Methods:**

A retrospective clinicopathological review of 10 histologically confirmed OANs was carried out. Whole exome sequencing (WES) of germline and paired tumour samples was performed for four of the ten OAN cases and compared to WES data from five cases of conventional ACC and data from The Cancer Genome Atlas. We reviewed all the cases of malignant OAN reported in the literature and compared to our case series.

**Results:**

Eight (80%) tumours were classified as malignant, one borderline and one benign (Lin–Weiss–Bisceglia criteria, LWB). The malignant OAN were larger tumours and had higher MIB index and Helsinki scores. Molecular profiling identified a pathogenic germline variant in *MSH6* in an individual in the OAN group. The OAN samples had a lower mutation burden compared to the ACC samples. Somatic driver variants were identified in OAN and ACC samples including a pathogenic missense variant in *CTNNB1*.

**Conclusion:**

In this study, the LWB classification demonstrated sensitivity for the differentiation of benign from malignant OAN. Molecular profiling identified dysregulation in DNA repair and Wnt signalling pathways in both OAN and ACC samples, suggesting a molecular overlap between OAN and conventional ACC.

## Introduction

Oncocytic adrenocortical neoplasm (OAN) represents a histological variant of adrenocortical neoplasm, consisting predominantly or exclusively of epithelial cells with abundant granular eosinophilic cytoplasm. The first adrenal oncocytoma was discovered in 1986 (Kakimoto *et al.* 1986), and since then, more than 150 cases have been reported in the literature (Peynirci * et al.* 2018).

Malignant OAN is reported to have a more favourable outcome with a superior 5-year survival rate compared to conventional ACC (Wong *et al.* 2011). Current prognostic scoring systems appear to overestimate the malignancy potential of OANs. The Weiss scoring system, which is widely used to distinguish benign from malignant adrenocortical neoplasms, cannot be applied to OAN, but three of the nine scoring criteria are intrinsic to OAN biology: high nuclear grade (<25% clear cells and diffuse architecture) (Bisceglia *et al.* 2004), automatically designating all OANs as malignant. The Lin–Weiss–Bisceglia (LWB) criteria, recommended by the 2017 WHO update on endocrine tumours (Lam 2017), categorizes an OAN as malignant if the tumour fulfils any of the following major criteria: mitotic rate >5 mitoses per 50 hpf, any atypical mitoses, any venous invasion or as borderline if the tumour exhibits any of the minor criteria: large size >10 cm and/or >200 g, necrosis, capsular invasion or sinusoidal invasion and benign if no major or minor criteria are fulfilled. Of recent interest, the Ki-67 proliferation index and the Helsinki score, a combination of morphological parameters (presence of necrosis and mitotic count >5 per 50 hpf) and Ki-67 index appear to be a more specific predictor of survival and poor outcome in malignant OAN (Duregon *et al.* 2017, Renaudin *et al.* 2018).

Molecular profiling provides key prognostic information in other endocrine tumours, and genetic profiling of adult-onset ACC has been investigated with the majority of cases occurring sporadically. However, cases have been identified associated with germline mutations in the mismatch repair genes *MSH6* and *MSH2* that cause Lynch syndrome (Raymond *et al.* 2013, Zheng *et al.* 2016, Casey *et al.* 2018). Additionally, a small subset of adult ACC cases (1–2%) is associated with mutations in multiple endocrine neoplasia type 1 gene *MEN1* (Else *et al.* 2014). Molecular and genetic studies of ACC have highlighted a number of key molecular predictors of oncogenic progression including *TP53*, insulin-like growth factor 2 (*IGF2*), multiple endocrine neoplasia type 1 (*MEN1*) and β-catenin (*CTNNB1*) (Giordano *et al.* 2003, Tissier *et al.* 2005, Zheng *et al.* 2016). Few molecular studies have included OAN, and therefore, little is known about the molecular profile of OAN compared to ACC.

The aims of this study were to describe and identify clinical, molecular and histopathologic markers of malignant vs benign OAN in our case series and in the literature in order to identify key prognostic markers, which may facilitate a stratified management approach for patients with OAN.

## Methods

### Clinical data

Data on 10 histologically confirmed OANs from an electronic hospital database in Cambridge University Hospitals NHS Trust, UK, from 2001 to 2018 were retrospectively reviewed. The secretory status of these adrenal tumours was guided by a standardised hospital-based biochemistry protocol consisting of plasma/urinary metanephrines, overnight dexamethasone suppression test/salivary cortisol/24 h urinary cortisol, ACTH, DHEAS, plasma aldosterone concentration and plasma renin activity.

Adrenal lesions were characterised as benign on CT if the Hounsfield unit (HU) on plain imaging was <10, absolute washout >60% or relative washout >40% or low signal intensity on out-of-phase signal images on MRI and indeterminate if these criteria were not fulfilled. Clinicopathological information including association with other malignancy, type of surgery, European Network for the Study of Adrenal Tumors (ENSAT) stage, treatment regimen and clinical outcome data were collected. Informed consent was obtained on all patients (IRAS ID 133065).

### Histopathological review

Histopathological examination was conducted by a single experienced pathologist with a special interest in endocrine pathology (AM). Tumour size, resection margins and tumour category were determined. R0 resection was defined as the absence of macroscopic or microscopic extension of the tumour at the surgical margin, while R1 resection was defined as the presence of microscopic extension of the tumour along the line of resection. Prognostic scoring systems including the Weiss and modified Weiss scores, LWB scores, Helsinki scores and Ki-67 proliferative index were recorded.

### Tumour immunohistochemistry

Immunohistochemical staining was performed with monoclonal mouse anti-human Ki-67 antibody (clone MIB-1, M7240; dilution 1:100; Agilent Technologies) in a Leica BOND-III IHC autostainer, with antigen retrieval performed at pH 9.0 by using a Leica Epitope retrieval solution 2 for 20 min at 100°C. Slides were counterstained with haematoxylin (Leica Biosystems) and mounted with ClearVue mounting medium (Thermo Fisher Scientific).

Ki-67 analysis was evaluated using manual analysis (MA) of 'hot spots' (Yamazaki *et al.* 2016). Hot spots, which contained the most frequent Ki-67-positive cells, were selected through scanning the whole immunostained slide preparation. MA was performed using the ×40 microscope objective of 1000 cells in hot spot areas. The Ventana Benchmark mismatch repair panel (MSH2 (G219-1129) and CONFIRM anti-MSH6) was performed on 4-µm sections of paraffin-embedded tumour tissue in accordance with the manufacturer’s guidelines and interpreted by an experienced pathologist (AM).

### Germline and tumour whole exome sequencing

Germline DNA was collected from four patients with OANs and five patients with ACC. Tumour DNA was analysed for four OAN cases and four ACC cases sequenced in house and reviewed alongside data available from The Cancer Genome Atlas (TCGA) for OANs (four cases) and ACC (86 cases). Germline DNA was extracted from blood. Tumour tissue was microdissected from formalin-fixed paraffin-embedded blocks, and DNA was extracted using a Covaris ultrasonicator. For all individuals, DNA was extracted from blood and prepared for PE125 whole exome sequencing (WES) using the Nextera Rapid Capture Exome enrichment kit (Illumina). Sequencing was performed on HiSeq-4000 machines and analysed with a standard in-house pipeline following GATK best practice recommendations for WES data (Supplementary material, see section on supplementary materials given at the end of this article). Germline TCGA data were downloaded in BAM format and reprocessed using the same WES pipeline (Supplementary material).

### Germline variant prioritisation and candidate selection

Germline sequencing data from all samples were analysed to identify any known cancer-predisposing variants. Common variants that appeared in greater than 1% of the non-Finnish European 1000 genomes population were removed from analysis. Protein-affecting variants (loss of function, inframe insertions and deletions and predicted deleterious and probably damaging missenses (as flagged by SIFT and PolyPhen respectively) were selected for further analysis. Variants that passed filtering were manually examined and prioritised to select candidates.

### Tumour variant analysis

A full set of somatic variants was generated for all nine tumour samples. Variants were filtered to select those with a variant allele frequency of greater than 10%. For each sample, tumour mutation burden was calculated. Hypermutated samples were defined as those with a mutation burden of greater than 10 mutations/Mb. Somatic variants were explored to identify any known links to tumour development. Copy number variants were assessed using CNVkit (Talevich *et al.* 2016), which was run in batch mode on matched tumour and normal samples.

Samples from TCGA were analysed in a separate batch to account for differences in exome capture and sequencing. The Log_2_ copy number ratio was assessed across binned regions for each matched pair and plotted to identify copy number changes. A gain of a single copy of a genomic region was defined by a log_2_ copy number ratio of 0.5, and a loss of a single copy was defined by a log_2_ copy number ratio of –1 (±25%) according to CNVkit protocol (Talevich *et al.* 2016). Metrics were calculated on copy number calls to filter out the false positives with a s.e. of greater than 0.01.

### Tumour mutational signatures

Tumour mutational signatures were calculated to identify any overlapping oncogenic drivers of ACC and OAN development. The deconstruct Sigs package for R was used to assess the pattern of somatic single nucleotide variants and assign to each sample the top COSMIC oncogenic signatures.

### Literature review

A comprehensive literature search was performed via PubMed using the MeSH terms 'adrenal or adrenocortical or adrenal gland' and 'oncocytic' and 'neoplasm or carcinoma or tumour' to retrieve all published full articles and abstracts related to OAN in English. Data extracted from the reports included age, gender, presentation, functionality, site, size and weight, prognostic scoring systems, Ki-67 proliferative index, treatment and clinical outcomes.

### Statistical analysis

Categorical variables were expressed as numbers (*n*) and percentages (%), and continuous variables were expressed as mean ± s.d. or median (minimum–maximum) if non-parametric. Data were analysed using SPSS version 23.0.

## Results

### Clinical features

The clinical characteristics of 10 OAN cases identified from our database are summarised in Table 1. According to the LWB criteria, eight cases were classified as malignant, one borderline and one benign. In the malignant category, six were males and two were females with a mean age of 60 ± 11 years. Three of eight cases were secretory adrenal tumours, of which two were cortisol secreting and the third was deoxycorticosterone secreting. Both cases in the borderline and benign category were cortisol secreting. Five of eight malignant OAN and one borderline OAN were characterised as indeterminate on imaging. The malignant OANs were larger with a mean maximum diameter of 120.7 ± 52.0 mm compared to the borderline (68 mm) and benign (20 mm) cases, respectively. Two patients with malignant OAN had a history of a second primary tumour, case 3 with a molecularly confirmed diagnosis of Lynch syndrome previously published by our group (Challis *et al.* 2016), and case 4 had a history of a metastatic well-differentiated neuroendocrine tumour of unknown primary. Finally, case 10 with a benign adrenal oncocytoma also had a history of a well-differentiated mid-gut neuroendocrine tumour.

**Table 1:** Clinical features and treatment of 10 oncocytic adrenocortical neoplasms prognosticated according to the LWB criteria system.

	Case 1^a^	Case 2	Case 3	Case 4	Case 5	Case 6	Case 7	Case 8	Case 9^a^	Case 10
LWB criteria	Malignant	Malignant	Malignant	Malignant	Malignant	Malignant	Malignant	Malignant	Borderline	Benign
Age (years)	66	43	55	71	58	72	71	46	59	64
Sex	M	M	F	M	M	M	M	F	F	F
Presenting feature	Incidental	Abdominal mass	Incidental	Incidental	Cushing’s syndrome, hypogonadism	Hypokalemia and proximal myopathy	Incidental	Incidental	Incidental	Incidental
Other malignancies	Nil	Nil	Ovarian carcinoma and a malignant colorectal polyp	Metastatic NET	Nil	Nil	Nil	Nil	Nil	Metastatic NET
Excess hormone	Cortisol	Nil	Nil	Nil	Cortisol	Deoxycorticosterone	Nil	Nil	Cortisol	Cortisol
Site	Left	Left	Right	Right	Left	Right	Left	Right	Left	Left
Max diameter (mm)	105	205	130	105	135	78	38	170	68	20
Imaging characterization	N/A	N/A	N/A	Indeterminate	Indeterminate	Indeterminate	Indeterminate	Indeterminate	Indeterminate	Benign
Type of surgery	Laparoscopic	Open and splenectomy	Open and nephrectomy	Open and liver resection	Laparoscopic	Open	Laparoscopic	Open	Laparoscopic	Open
Weight (g)	328	1893	927	N/A	214	240	63.5	968	60	23
Adjuvant treatment	Mitotane + EDP	Mitotane + RT+ EDP	Mitotane	Nil	Mitotane	Nil	Nil	Nil	Nil	Nil

^a^Referred postoperatively to our centre.

EDP, etoposide, doxorubicin, carboplatin; NET, Neuroendocrine tumour; RT, radiotherapy.

### Immunohistopathological review

The histological findings are summarised in Table 2. All cases in the malignant group had a Weiss and modified Weiss score of >3 compared to the borderline/benign group with scores of <3. Seven out of eight malignant OAN cases were pure oncocytic tumours (composed of at least 90% oncocytes). All but one case (case 4) in the malignant category of OANs were classified as ENSAT stage 3 or above at presentation compared to the borderline/benign OAN group, which were classified as ENSAT stage 2. The malignant OANs had a median Ki-67 proliferative index of 16.45% (range: 3–53%) and median Helsinki score of 22.1 (range: 8–61.3) greater than that of the borderline (Ki-67 proliferative index 1.3 and Helsinki score 1.3) and benign (Ki-67 proliferative index 2.1 and Helsinki score 2.1) cases. The three malignant OAN cases with poorer outcomes (cases 1, 2, 5) had a greater average Ki-67 proliferative index of 27.9 and Helsinki score of 34.9 when compared to the five cases with better outcomes (Ki-67 proliferative index of 13.4 and Helsinki score 18 but were not statistically significant, *P* = 0.215, *P* = 0.161). MSH2 and MSH6 staining were absent in case 3 and a similar staining pattern was identified in the OAN from case 2, although no pathogenic germline or somatic variant in a mismatch repair gene was identified.

**Table 2:** Immunohistopathological features and outcome of 10 oncocytic adrenocortical neoplasm.

	Case 1^a^	Case 2	Case 3	Case 4	Case 5	Case 6	Case 7	Case 8	Case 9^a^	Case 10
Weiss criteria	8	7	6	6	6	6	3	6	2	1
Modified Weiss criteria	7	5	5	4	7	6	3	4	1	2
LWB criteria	Malignant	Malignant	Malignant	Malignant	Malignant	Malignant	Malignant	Malignant	Borderline	Benign
Category	Pure	Pure	Pure	Pure	Mixed	Pure	Pure	Pure	Mixed	N/A
Resection margin	R1	R1	R0	R0	R1	R0	R0	R0	R1	R0
Ki-67 (%)	13.5	16.9	17.4	8.2	53.3	16	22.8	3	1.3	2.1
Helsinki score	21.5	21.9	22.4	13.2	61.3	24	22.9	8	1.3	2.1
ENSAT stage	3	3	3	2	3	3	2	2	2	N/A
MSH 2	Preserved	Absent	Absent	Preserved	Preserved	Preserved	Preserved	Preserved	Preserved	N/A
MSH 6	Preserved	Absent	Absent	Preserved	Preserved	Preserved	Preserved	Preserved	Preserved	N/A
Recurrence/metastases	Recurrence locally, peritoneum and rectum	Bone metastases	Nil	Nil	Recurrence locally & peritoneum	Nil	Nil	Nil	Nil	Nil
Outcome	DOD at 24 months	AED at 82 months	ANED at 232 months	ANED at 87 months	AED 38 months	ANED at 25 months	ANED at 43 months	ANED at 60 months	ANED at 32 months	ANED at 96 months

^a^Referred postoperatively to our centre.

AED, alive with evidence of disease; ANED, alive with no evidence of disease; DOD, dead of disease.

### Disease outcomes

All individuals with malignant OAN cases had adrenalectomy, three laparoscopically and five open, achieving R0 resection in a third laparoscopically and 80% open. The three cases with R1 resection margins (two laparoscopic adrenalectomy and one open adrenalectomy) had poor outcomes including one death following tumour recurrence (case 1) and two cases with metastases or recurrence with residual disease (cases 2 and 5). The median follow-up was 51.5 (range: 24–232) months. The five remaining individuals with malignant OAN remain free of disease with follow-up period from 25 to 232 months (Table 1).

### Germline whole exome sequencing analysis

A germline, loss of function, splice donor variant (NM_000179.2:c.3438+1G>A, rs267608096) in the Lynch syndrome gene *MSH6* was identified in one affected female (case 3). Immunohistochemistry studies on available tumour showed a loss of mismatch repair proteins MSH2 and MSH6. This variant has previously been described by our group and has been labelled as a ‘likely pathogenic’ Lynch syndrome variant (ClinVar submission accession: SCV000108066.2). Immunohistochemistry of the tumour from a second individual with OAN (case 2) also demonstrated loss of *MSH2* and *MSH6* expression; however, no germline predicted pathogenic variants in the Lynch syndrome genes were identified.

Comparing the results of the germline sequencing data from the OAN group with the five cases of conventional ACC, a second pathogenic germline Lynch syndrome-associated variant was identified in a female in the conventional ACC group (ACC6), which has also previously been published by our group (Casey *et al.* 2018) (Table 3). This was a frameshift deletion (NM_000251:c.787delA) in MSH2 which was consistent with the loss of *MSH2* and *MSH6* expression seen in the tumour and a medical history consistent with Lynch syndrome (Casey *et al.* 2018). Reviewing the TCGA-ACC dataset, no pathogenic or likely pathogenic variants in *MSH2*, *MSH6* or *MSH3* were identified. However, predicted deleterious and damaging missense variants were found in mismatch repair genes *MLH1* and *PMS2.* A missense variant in *MLH1* (NM_000249.2c.1853A>C) was found in two individuals, both with conventional ACC. The missense variant in *PMS2* (NM_000535.7:c.903G>T) was reported as 'Likely Pathogenic' by ClinSig and was found in one individual with conventional ACC.

**Table 3:** Malignant oncocytic adrenocortical neoplasms: review of the literature and clinicopathologic data and outcomes.

Total cases	Author, year	*N*	Age	Gender	Presentation	Functionality	Site	Size (mm)	Weight (g)	Category	Mibi	Recurrence/metastases	Outcome	Mitotane	Chemotherapy/radiotherapy
1	(el-Naggar *et al*. 1991)	1	56	M	Abdominal pain	No	R	80	N/A	N/A	N/A	N/A	N/A	Yes	Radiotherapy
2	(Kurek *et al.* 2001)	1	74	F	Incidental	No	L	N/A	N/A	N/A	N/A	Recurrence (local) and metastases (Ovarian)	AED	No	No
6	(Hoang *et al.* 2002)	1	39	M	Ascites, abdominal pain	No	L	140	N/A	N/A	N/A	N/A	ANED	No	No
		2	53	F	Abdominal pain	No	L	170	1200	N/A	N/A	Metastases (bone and lung)	AED	No	No
		3	58	M	Abdominal mass	No	R	130	740	N/A	N/A	No	ANED	No	No
		4	71	M	Abdominal mass	No	L	85	100	N/A	N/A	No	ANED	No	No
7	(Tanaka *et al.* 2004)	1	54	M	Abdominal mass	Subclinical CS	R	N/A	N/A	N/A	N/A	Metastases (Bone, lung, liver, contralateral adrenal)	AED	Yes	No
8	(Seo *et al.* 2002)	1	49	F	Incidental	No	L	85	N/A	N/A	N/A	Recurrence (Intrabdominal carcinomatosis)	DOD	No	No
12	(Song *et al.* 2004)	1	64	F	Incidental	No	L	110	420	N/A	<1	No	ANED	No	No
		2	47	F	Abdominal pain	No	L	150	1220	N/A	<1	Recurrence and metastases (retroperitoneal mass and celiac lymph node)	ANED	No	No
		3	37	F	Abdominal pain	No	L	115	410	N/A	<1	No	ANED	No	No
		4	35	M	Incidental	No	R	85	290	N/A	<1	No	ANED	No	No
16	(Bisceglia *et al.* 2004)	1	46	M	Gynecomastia	No	R	170	1900	N/A	5–20	Recurrence	DOD	N/A	N/A
		2	32	F	Incidental	Subclinical CS	R	110	2520	N/A	2–15	Recurrence	AED	N/A	N/A
		3	62	F	Incidental	No	R	80	260	N/A	5	No	DNOD	N/A	N/A
		4	77	F	Incidental	No	L	100	120	N/A	5–20	No	ANED	N/A	N/A
17	(Ali & Raphael 2007)	1	25	M	Hypokalemia	Cortisol and aldosterone secreting	R	85	90	N/A	10	Recurrence (local and hepatic invasion)	AED	N/A	N/A
18	(Ohtake *et al.* 2010)	1	69	M	Abdominal pain	No	L	75	N/A	N/A	5.9	N/A	N/A	N/A	N/A
19	(Argyriou *et al.* 2008)	1	54	M	Lung mass	No	R	N/A	N/A	N/A	10–20	Recurrence and metastases (lung and bone)	DOD	Yes	Chemotherapy
20	(Juliano *et al.* 2008)	1	45	F	Abdominal pain	Androgen excess	R	110	410	N/A	N/A	Recurrence and metastases (bone, liver, lung)	AED	No	Chemotherapy & radiotherapy
21	(Mwandila *et al.* 2010) (Abstract)	1	19	F	Hirsutism, acne, oligomenorrhea	Androgen excess	L	50	N/A	N/A	N/A	N/A	N/A	N/A	N/A
29	(Wong *et al.* 2011)	1	53	F	Virilizing	Androgen excess	L	130	670	Pure	5	Recurrence and metastases	AED	N/A	N/A
		2	36	F	Incidental	No	L	145	885	Pure	30	N/A	DOD	N/A	N/A
		3	69	F	Incidental	No	L	60	76	Pure	2	N/A	N/A	N/A	N/A
		4	47	F	Incidental	Androgen excess	L	105	552	Pure	30	N/A	DOD	N/A	N/A
		5	36	M	CS	Cortisol excess	R	80	155	Pure	10	N/A	ANED	N/A	N/A
		6	41	F	Virilizing	Androgen excess	L	285	5720	Pure	6	Metastases (liver)	AED	N/A	N/A
		7	68	F	Incidental	Cortisol excess	R	80	70	Pure	30	Recurrence	DOD	N/A	N/A
		8	29	M	Gynecomastia	Estrogen/prolactin excess	L	200	1120	Pure	10	Recurrence	AED	N/A	N/A
41	(Duregon *et al.* 2011)	1	31	F	N/A	No	L	95	255	Pure	N/A	N/A	ANED	N/A	N/A
		2	60	M	N/A	No	L	16	8	Pure	N/A	N/A	ANED	N/A	N/A
		3	68	F	N/A	No	R	170	N/A	Pure	N/A	N/A	ANED	N/A	N/A
		4	66	F	N/A	No	N/A	N/A	N/A	Pure	N/A	N/A	ANED	N/A	N/A
		5	46	M	N/A	No	L	180	950	Pure	N/A	N/A	ANED	N/A	N/A
		6	32	M	N/A	No	R	230	N/A	Pure	N/A	N/A	DOD	N/A	N/A
		7	44	F	N/A	Cortisol excess	R	80	N/A	Mixed	N/A	N/A	DOD	N/A	N/A
		8	35	M	N/A	No	L	80	40	Mixed	N/A	N/A	ANED	N/A	N/A
		9	67	F	N/A	Cortisol excess	L	150	1050	Mixed	N/A	N/A	ANED	N/A	N/A
		10	44	M	N/A	Cortisol excess	R	200	1300	Mixed	N/A	N/A	DOD	N/A	N/A
		11	46	F	N/A	No	L	99	270	Mixed	N/A	N/A	ANED	N/A	N/A
		12	28	M	N/A	Cortisol excess	L	110	210	Mixed	N/A	N/A	DOD	N/A	N/A
42	(Kalra *et al.* 2015)	1	34	M	Incidental	No	L	160	N/A	N/A	N/A	No	ANED	N/A	N/A
43	(Carré *et al.* 2016)	1	50	F	Virilizing	Androgen	L	35	N/A	N/A	6.8	No	ANED	N/A	N/A
44	(Sumner *et al.* 2017) (Abstract)	1	83	F	Incidental	No	N/A	N/A	N/A	N/A	N/A		N/A	N/A	N/A
45	(Panizzo *et al.* 2018)	1	48	M	Incidental	No	L	123	300	N/A	20	No	ANED	No	N/A
46	(Al Balooshi *et al.* 2018)	1	37	M	Incidental	Cortisol	L	N/A	N/A	N/A	N/A		N/A	N/A	N/A
80	(Renaudin *et al.* 2018)	34	50 (42–57)	19 F (55%)	Incidental 16 (47%)	17 (54%)	24 L (70%)	83 (60–126)	200 (80–500)	2/3 pure	Poor outcome (*n* = 3) 12 Good outcome (*n* = 23) 3	3 Recurrence	2 died 1 AED	18 (55%)	4 (Radiotherapy)

AED, alive with evidence of disease; ANED, alive with no evidence of disease; DOD, dead of disease.

### Tumour whole exome sequencing analysis

Whole exome sequencing of DNA extracted from tumour was performed for two OAN samples and analysed in conjunction with sequence data from four OAN samples from the TCGA cohort and compared to three ACC tumour samples sequenced in house. In total, 2325 somatic variants were called across the set (542 synonymous, 1783 non-synonymous); 78% of these (1828 variants) were found in two hypermutated tumours, one oncocytic and one conventional ACC tumour. One of these hypermutated tumours with a mutation burden of 14.97 mutations/Mb was from case 3 with a pathogenic germline *MSH6* variant. This is consistent with previous evidence that Lynch-driven ACCs have a higher mutation burden than non-Lynch tumours (Zheng *et al.* 2016). The average mutation burden in this cohort was 6.35 mutations/Mb and was markedly higher for in-house sequenced OAN samples (10.67 mutations/Mb) than for the included OAN TCGA samples (0.95 mutations/Mb). On average, the ACC samples had a higher mutation burden than the OAN samples (usual type ACC: 11.72 mutations/Mb, OAN: 3.67 mutations/Mb) although these results are largely driven by the presence of the two hypermutated samples. Of the non-hypermutated samples, the average mutation burden was 1.70 mutations/Mb and was comparable between ACC and OAN samples (ACC: 2.45 mutations/Mb, OAN: 1.41 mutations/Mb).

A frameshift deletion in *ATM* (NM_000051:c.1875delT) (VAF 41.0%) and a pathogenic missense variant in *TP53* (NM_000546.5:c.524G>A, rs28934578) (VAF 27.9%) were identified in the OAN from case 3 (Table 3). Other notable findings in this tumour included missense variants in *NFKB2*, *NF2* and *USP6*. We also identified a pathogenic missense variant in *CTNNB1* in the OAN of case 1 with a VAF of 42.6%.

Of the four OAN tumour samples from TCGA, a somatic pathogenic mutation within a driver gene was found in only one sample. An OAN carried a somatic *PIK3CA* missense variant (NM_006218.2:c.3140A>G, rs121913279) with a VAF of 21.0% (Table 4). This OAN had the highest mutation burden of the tested TCGA samples with 1.51 mutations/Mb.

**Table 4:** Pathogenic Germline candidate variants identified in cases with OAN and compared to ACC cases and TCGA-ACCA data set.

Case	Sex	Tumour type	Tumour tissue sequenced	Mutations per megabase in tumour	Germline variant
Case 1	Male	OAN	Yes	3.243979	NA
Case 2	Male	OAN	No	NA	NA
Case 3	Female	OAN	Yes	14.96623	MSH6:NM_000179.2:c.3438+1G>A
Case 7	Male	OAN	No	NA	NA
Case 8	Female	OAN	No	NA	NA
ACC1	Female	ACC	Yes	2.637655	NA
ACC2	Male	ACC	Yes	2.26337	NA
ACC3	Female	ACC	No	NA	NA
ACC4	Male	ACC	No	NA	NA
ACC 5	Female	ACC	Yes	30.26555	NA
ACC6	Female	ACC	No	NA	MSH2:NM_000251:c.787delA
TCGA_OR_A5JD	Female	OAN	Yes	0.981044	NA
TCGA_OR_A5JH	Female	OAN	Yes	0.424729	NA
TCGA_OR_A5K3	Male	OAN	Yes	0.886876	NA
TCGA_OR_A5LK	Male	OAN	Yes	1.509795	NA

Somatic copy number alterations were noted in one OAN sample (case 1) compared to three conventional ACC samples. Finally, mutation signature analysis was performed to identify the predominant mutational processes enacting on these tumours. COSMIC signature 1 was the most commonly occurring signature across all samples. The presence of this signature correlates with age and occurs in most cancer samples according to the COSMIC-predicted aetiology (Alexandrov *et al.* 2013). There was no evidence of a difference in mutational signatures between OAN and conventional type ACC samples in this study.

### Literature review

Eighty malignant OAN cases described in 18 full English articles and two abstracts were reviewed and detailed in Table 5. Malignant OANs occur at a mean age of 49 ± 15 years with a female predilection in a 1.2:1 ratio. The neoplasms were large with a mean maximum diameter of 117.7 mm ± 53.4 mm. From the available data, 45% of tumours were secretory; 42.8% of malignant OANs were treated with mitotane, 10.4% with radiotherapy and 2% with chemotherapy alone or in combination with radiotherapy. A total of 16.2% of patients died as direct cause of their OAN, 13.5% of patients were alive with residual disease from either recurrence or metastases and 68.9% were disease free and well. The most common site of recurrence/metastases was bone, lungs, liver, adrenal bed, peritoneum, ovary and contralateral adrenal gland. The clinical and pathological data and outcomes were compared to our case series detailed in Supplementary Table 1.

**Table 5:** Somatic variants identified in ACC tumours. Oncocytic ACCs are indicated with *.

Gene	Transcript ID	Variant	ID	Consequence	Sample	Variant allele frequency
*APC*	NM_000038	c.4718delA	COSM267970	Frameshift variant	ACC1	0.78
*ATM*	NM_000051	c.1875delT	COSM1350784	Frameshift variant	Case 3*	0.41
*CTNNB1*	NM_001330729.2	c.113C>T	rs121913407	Missense variant	Case 1 and ACC2	ACC1:0.43, ACC4:0.42
*MSH6*	NM_000179	c.846delG	NA	Frameshift variant	ACC2	0.17
*NF2*	NM_000268	c.1231C>T	rs773296925	Missense variant	Case 3*	0.29
*NFKB2*	NM_001077494	c.2369G>A	NA	Missense variant	Case 3*	0.22
*PIK3CA*	NM_006218.2	c.3140A>G	rs121913279	Missense variant	TCGA_OR_A5LK*	0.21
*TP53*	NM_000546.5	c.524C>T	rs28934578	Missense variant	Case 3*	0.28
*TP53BP1*	NM_005657	c.4339G>A	COSM961975	Stop gained	ACC5	0.14
*USP6*	NM_004505	c.2197C>T	NA	Missense variant	Case 3*	0.34

## Discussion

OANs are rare tumours with an indolent nature conferring a superior clinical outcome and survival rate compared to conventional ACC. Estimated overall median survival for malignant OAN is 58 months compared to conventional ACC, which is 14–32 months (Wong *et al.* 2011), and survival at 2 years for malignant OAN surpasses 92% compared to 61% in conventional ACC (Renaudin *et al.* 2018).

A complete, margin-free surgical resection is crucial in determining positive clinical outcomes for ACC and malignant OANs. Achieving R0 resection in adrenocortical carcinoma compared to R1 resection improved 5-year overall survival rates (64.8% vs 33.8%, *P* < 0.001) and 5-year recurrence-free survival rates (30.3% vs 13.8%, *P* = 0.03) with surgical margin status as an independent predictor of poor overall survival (Margonis *et al.* 2016). The three malignant OANs (two laparoscopic adrenalectomy and one open adrenalectomy) with poor outcomes resulting in one death and two metastatic recurrence in our series had a R1 resection margin (Table 1). Although data on resection margins for malignant OAN described in case reports/series were scarce, Renaudin *et al*. reported that all 43 malignant OANs in their series underwent complete surgical treatment with R0 resection and only three poor outcomes ensued: two deaths and one alive with relapse (Renaudin *et al.* 2018). In a recent review, the surgical approach to adrenal cancers (ENSAT I-III) either by laparoscopic or open adrenalectomy confers similar R0 resection rates, overall recurrence, disease free and overall survival rates implying that the adequacy of tumour resection is pivotal rather than the surgical approach (Mpaili *et al.* 2018).

Four cases of malignant OANs in our series were treated with mitotane achieving therapeutic mitotane levels comparable with the literature with almost half of malignant OAN cases treated with mitotane. The overall survival in the mitotane-treated malignant OAN (in comparison to the mitotane-treated conventional ACC group) was better, possibly due to the unique biological behaviour of OAN (Renaudin *et al.* 2018). Experience with other treatment modalities such as chemotherapy and radiotherapy in our series and in the literature is limited.

Following the recommendation by the WHO of endocrine tumours (Lam 2017), we classified our cohort according to the LWB score into malignant, borderline and benign category, and we also applied the Helsinki score and reviewed the proliferation indices. Our cases from the borderline and benign category of OANs had low proliferation indices <5% and the remaining malignant cases except one (case 8: 3%) had indices >5% (Table 2). Bisceglia *et al*. documented that a proliferation index of ≥5% correlated well with mitotic activity in all malignant cases including recurrences for both ACC and OANs, except in a single case with a value <2%, and all benign tumours in this study had a proliferation index <5% (range 0–4%) (Bisceglia *et al.* 2004).

The Helsinki score, consisting of a combined assessment of morphological indices (mitotic count >5 per hpf and presence of necrosis) and Ki-67 proliferation index, appears to be the most specific in predicting the clinical outcome of malignant OAN. Initially introduced as a prognostic assessment system for ACC, a score of 8.5 was able to identify metastatic ACC (Pennanen *et al.* 2015), and in a more recent paper, a Helsinki score >8.5 appears to be most discriminative in identifying aggressive malignant OAN with a better specificity compared to the LWB score, Weiss score and reticulin algorithm (Renaudin *et al.* 2018). Seven out of eight cases in our malignant OAN series had a Helsinki score of >8.5, including the three cases for whom poor outcomes were recorded with scores of 21.5, 21.9 and 61.3, respectively. The two cases in the borderline and benign category had low scores of 1.3 and 2.1, respectively. Although Ki-67 proliferative index incorporated into the Helsinki score may play a role in predicting poorer outcomes, the presence of necrosis, another variable in the score, was the only histological difference between malignant OAN with poor outcomes vs those with good outcomes in the largest series of malignant OAN to date (Renaudin *et al.* 2018). In this smaller study, necrosis was identified at histological review in five malignant OANs from patients who have had a good outcome to date with no evidence of disease recurrence or metastases at a median of 60 months post-surgery.

Germline and somatic pathogenic variants were identified in *MSH2* and *MSH6*, further highlighting the role of mismatch repair genes in both conventional ACC and OANs. An additional case with a malignant OAN in this series (case 2) had evidence of mismatch repair deficiency on immunohistochemistry (loss of MSH2 and MSH6 expression) but no pathogenic germline or somatic variant was identified. The OAN from case 3 was found to be hypermutated and also had heterozygous somatic variants in DNA repair and checkpoint genes *TP53* and *ATM,* which potentially contributed to the hypermutation phenotype.

Neither of the main oncogenic events identified in this study, including DNA repair deficiency and dysregulation of Wnt signalling by β-catenin, were specific to usual-type or oncocytic-type ACCs. Patients with both tumour types had a comparable age of onset and were equally affected by germline predisposing variants in mismatch repair genes. Similarly, there was little difference in mutation burden of both tumour types, and no defining mutation signatures that could be attributed to each group were identified. This suggests that the oncocytic subtype of ACC is genetically similar to conventional ACC in both predisposing and oncogenic mechanisms. It seems likely that OANs will have similar molecular drivers to those described by the TCGA-ACC study (Zheng *et al.* 2016).

Limitations to this study include the retrospective design and small study number, but the study was enriched through inclusion of an up-to-date literature review and by comparing sequencing data from this series to data available in TCGA.

In summary, the LWB classification demonstrated sensitivity for the differentiation of benign from malignant OAN in this series. A proliferation index >5% and Helsinki score >8 also predicted poorer outcomes for malignant OANs in this series, and these scores may aid in stratifying the management of OANs in clinical practice. Ensuring a complete, margin-free resection is crucial in improving overall prognosis and survival. Finally, molecular profiling identified dysregulation in DNA repair and Wnt signalling pathways in both OAN and ACC samples, suggesting a shared molecular overlap between OAN and conventional ACC.

## Supplementary Material

SUPPLEMENTARY TABLE 1: COMPARING CLINICAL FEATURES AND OUTCOMES BETWEEN LOCAL CASE SERIES AND REPORTED CASES IN THE LITERATURE

SUPPLEMENTARY TABLE 2

Supplementary Figure 1

Supplementary Figure 2

## Declaration of interest

The authors declare that there is no conflict of interest that could be perceived as prejudicing the impartiality of the research reported.

## Funding

Dr Casey is funded by GIST Support UK. This work has been funded and supported by the UK Medical Research Councilhttp://dx.doi.org/10.13039/501100000265/Sackler programme (E F), the European Union Seventh Framework Program (2007–2013)/European Research Councilhttp://dx.doi.org/10.13039/501100000781 (310018) (M T).

## Author contribution statement

E F, S K, R C, B C, V K, M T, O G, A S, A L, G R C and A M were involved in study design and concept and drafting and review of the final manuscript. E F, S K, R C, G R C and A M were involved in data collection and analysis and E F, S K, M T, B C, A M, A S, V K and A L contributed to the final review of data included.
