# Supplementary material for: Investigating the clinical, pathological and molecular profile of oncocytic adrenocortical neoplasms: a case series and literature review
Source: Endocr Oncol. 2021 Aug 16;1(1):33–44. doi: 10.1530/EO-21-0011 (PMC10265542; doi:10.1530/EO-21-0011)
Supplement: SUPPLEMENTARY TABLE 1: COMPARING CLINICAL FEATURES AND OUTCOMES BETWEEN LOCAL CASE SERIES AND REPORTED CASES IN THE LITERATURE [file supplementary_table_1.pdf]

SUPPLEMENTARY TABLE 1: COMPARING CLINICAL FEATURES AND OUTCOMES BETWEEN LOCAL CASE SERIES AND REPORTED CASES IN THE LITERATURE		
	Case Series<br>*n*=8	Literature<br>*n*=80
Age (yr)	60 ± 11	49 ± 15
Sex , *n* (%)<br>Male	 6 (75.0)	 36 (45.0)
Hormonal excess, *n* (%)	3 (37.5)	34 (42.5)
Site, *n* (%)<br>Left	 4 (50.0)	*n*=78<br>52 (66.7)
Size (mm)	120.7 ± 52.0	*n*=74<br>117.7 ± 53.43
Weight (g)	328 (63.5-1893)	*n*=64<br>410 (8-5720)
Mitotane, *n*, (%)	4 (50.0)	21 (42.8)
Radiotherapy alone *n*, (%)<br>Chemotherapy alone *n*, (%)<br>Radiotherapy & Chemotherapy *n*, (%)	0<br>1 (12.5)<br>1 (12.5)	*n*= 48<br>5 (10.4)<br>1 (2.0)<br>1 (2.0)
Recurrence/Metastases, *n*, (%)<br>Yes	 3 (37.5)	*n*= 58<br>17 (29.3)
Outcome, *n* (%)<br>DOD<br>AED<br>ANED<br>DNOD	 1 (12.5)<br>2 (25.0)<br>5 (62.5)<br>0 (0.0)	*n*= 74<br>12 (16.2)<br>10 (13.5)<br>51 (68.9)<br>1 (1.3)

DOD= Dead of disease, AED= Alive with evidence of disease, ANED= Alive with no evidence of disease, DNOD=Died not of disease
