# Supplementary figures and images for: Investigating the clinical, pathological and molecular profile of oncocytic adrenocortical neoplasms: a case series and literature review
Source: Endocr Oncol. 2021 Aug 16;1(1):33–44. doi: 10.1530/EO-21-0011 (PMC10265542; doi:10.1530/EO-21-0011)

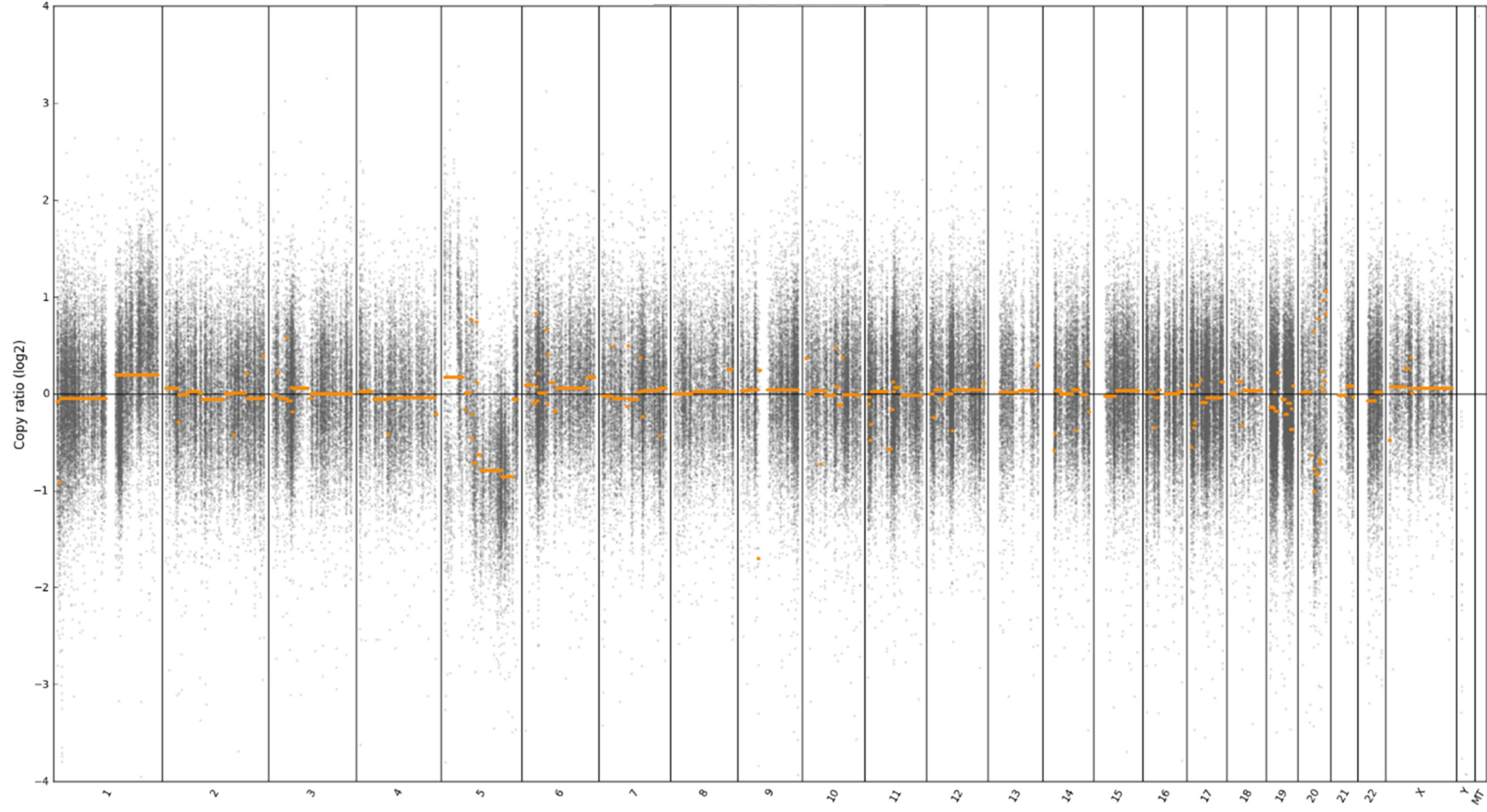

Supplement: Supplementary Figure 1 [file supplementary_figure_1.pdf]

**A**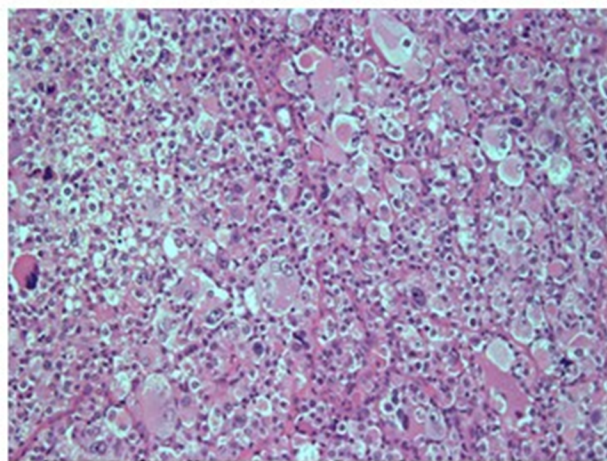**B**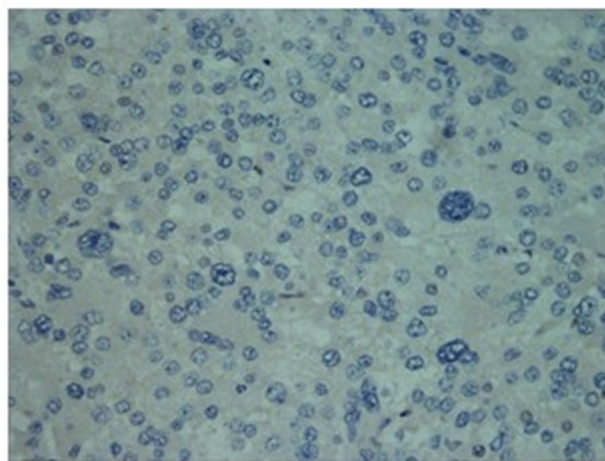**C**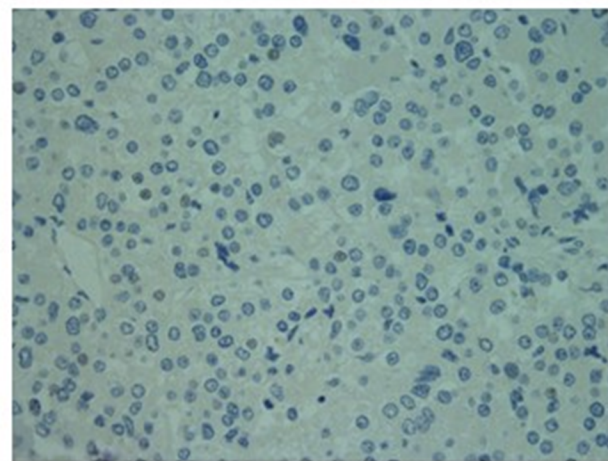**D**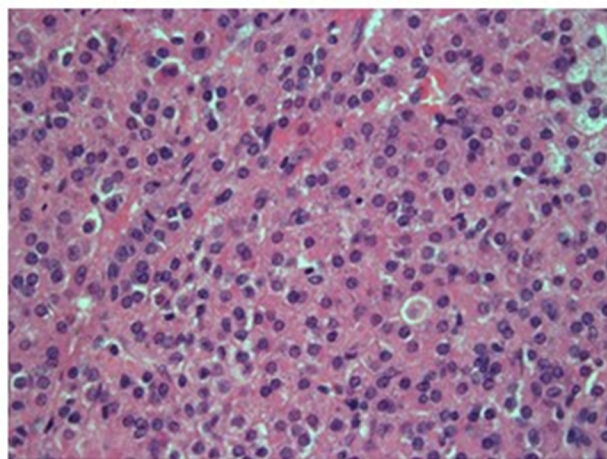**E**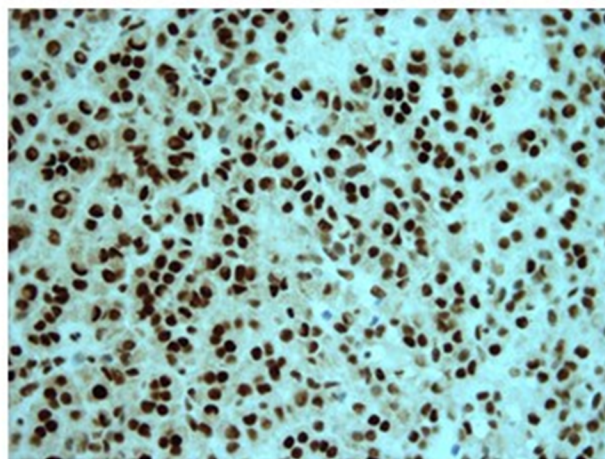**F**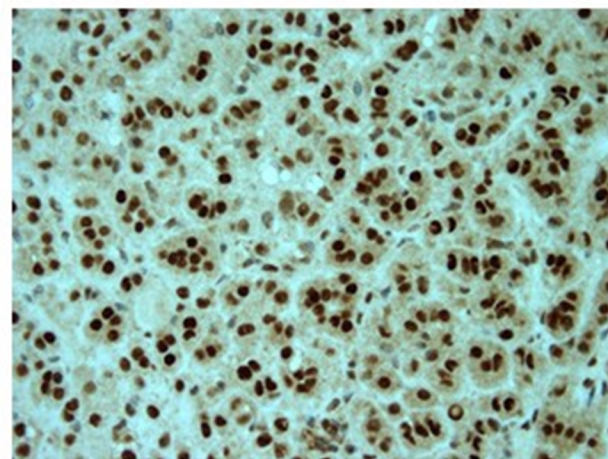

Supplement: Supplementary Figure 2 [file supplementary_figure_2.pdf]
